# Shared Components of Rhythm Generation for Locomotion and Scratching Exist Prior to Motoneurons

**DOI:** 10.3389/fncir.2017.00054

**Published:** 2017-08-11

**Authors:** Zhao-Zhe Hao, Ari Berkowitz

**Affiliations:** ^1^Department of Biology, University of Oklahoma, Norman OK, United States; ^2^Cellular and Behavioral Neurobiology Graduate Program, University of Oklahoma, Norman OK, United States

**Keywords:** central pattern generator, spinal cord, behavioral choice, synaptic input, motoneuron, turtle

## Abstract

Does the spinal cord use a single network to generate locomotor and scratching rhythms or two separate networks? Previous research showed that simultaneous swim and scratch stimulation (“dual stimulation”) in immobilized, spinal turtles evokes a single rhythm in hindlimb motor nerves with a frequency often greater than during swim stimulation alone or scratch stimulation alone. This suggests that the signals that trigger swimming and scratching converge and are integrated within the spinal cord. However, these results could not determine whether the integration occurs in motoneurons themselves or earlier, in spinal interneurons. Here, we recorded intracellularly from hindlimb motoneurons during dual stimulation. Motoneuron membrane potentials displayed regular oscillations at a higher frequency during dual stimulation than during swim or scratch stimulation alone. In contrast, arithmetic addition of the oscillations during swimming alone and scratching alone with various delays always generated irregular oscillations. Also, the standard deviation of the phase-normalized membrane potential during dual stimulation was similar to those during swimming or scratching alone. In contrast, the standard deviation was greater when pooling cycles of swimming alone and scratching alone for two of the three forms of scratching. This shows that dual stimulation generates a single rhythm prior to motoneurons. Thus, either swimming and scratching largely share a rhythm generator or the two rhythms are integrated into one rhythm by strong interactions among interneurons.

## Introduction

The central nervous system (CNS) can generate many rhythmic behaviors appropriately even without movement-related sensory feedback. How is the CNS organized to generate these different rhythms? Are different rhythmic behaviors involving the same motoneurons and muscles generated by the same set of neurons or different sets?

In some invertebrate cases, two different rhythmic behaviors are generated by completely separate neuronal networks (Heitler, 1985; Ramirez and Pearson, 1988; Hennig, 1990). In other cases, two rhythmic behaviors are generated by one shared network (Jing and Weiss, 2001; Kupfermann and Weiss, 2001; Marder and Bucher, 2001) or by partly shared networks (Briggman and Kristan, 2008).

Vertebrate interneurons that are rhythmically active during multiple rhythms have been found for cat locomotion and scratching (Geertsen et al., 2011; Trejo et al., 2015), rat locomotion and respiration (Le Gal et al., 2016), turtle swimming and scratching (Berkowitz, 2002, 2005, 2008, 2010), and tadpole and larval zebrafish swimming and struggling (Soffe, 1993; Li et al., 2007; Liao and Fetcho, 2008). One limitation of single-neuron recording in large vertebrate networks, however, is that an individual cell’s effect on the network’s output is generally so small that it is hard to demonstrate by selectively activating or silencing the neuron. Thus, one cannot conclude definitively for any individual interneuron recorded in such a large network that it is part of the central pattern generator for one or more rhythmic behaviors. In addition, vertebrate behaviorally specialized interneurons have also been demonstrated, including tadpole struggle-specialized neurons (Li et al., 2007; Li, 2015), zebrafish struggle-, or escape-specialized neurons (McLean et al., 2007, 2008; Liao and Fetcho, 2008; Satou et al., 2009), and turtle scratch-specialized neurons (Berkowitz, 2002). Thus, it is not clear from these studies to what extent rhythm-generating networks are shared.

Another approach to understand the organization of vertebrate rhythm-generating networks is to activate simultaneously the networks that generate two different motor patterns and assess network interactions via motor output (Carter and Smith, 1986; Stein et al., 1986; Currie and Stein, 1988; Ramirez and Pearson, 1988; Hennig, 1990; Svoboda and Fetcho, 1996; Earhart and Stein, 2000; Juranek and Currie, 2000; Berkowitz and Hao, 2011; Hao et al., 2011; Pujala et al., 2016). If activation of one network perturbs the rhythm and/or pattern of the other network, this demonstrates sharing of network elements and/or strong interactions between the networks. The turtle spinal cord contains sufficient circuitry to generate several rhythmic hindlimb motor patterns without brain inputs or movement-related sensory feedback. These motor patterns include forward swimming and three forms of scratching (rostral, pocket, and caudal) (Stein, 2005). These motor patterns can be reliably evoked by electrical or mechanical stimulation, respectively. Simultaneous swim and scratch stimulation can alter the motor patterns in multiple ways. For instance, when combined with a scratch stimulation, (1) a suprathreshold swim stimulation can trigger a rhythm with a cycle frequency higher than either swimming or scratching alone, (2) a suprathreshold swim stimulation can cause switches between swimming and scratching, and (3) an overly high-frequency swim stimulation can evoke a normal swimming motor pattern even though the swim stimulation by itself evokes only tonic hip-extensor activity (Hao et al., 2011).

These changes in cycle frequency and motor patterns demonstrate that scratch-evoking stimulation can influence both the rhythm and the pattern of swimming, and vice versa. But these results do not indicate whether the interactions occur at the level of interneurons or motoneurons. Traditionally, motoneurons have been viewed as passive recipients of premotor inputs, stemming from Sherrington’s idea of motoneurons as the final common path for motor commands (Sherrington, 1906). However, motor neurons have been shown to play key roles in rhythm generation in several invertebrates (Marder and Bucher, 2001). In vertebrates, motoneurons also have active properties that can shape rhythmic output to muscles (Hounsgaard and Kiehn, 1989; Kiehn et al., 2000; Heckman et al., 2008; Hultborn et al., 2013) and may contribute to rhythm generation via synapses with interneurons (Perrins and Roberts, 1995; Kiehn et al., 2000; Tresch and Kiehn, 2000; Mentis et al., 2005; Song et al., 2016; Lawton et al., 2017). If motoneurons themselves integrate two rhythmic inputs to produce one rhythmic output, this would add to the evidence that vertebrate motoneurons participate in rhythm generation.

Here, we recorded intracellularly from motoneurons that are involved in the two motor patterns *in vivo* while delivering swim and scratch stimulation simultaneously (“dual stimulation”) to trigger the effects mentioned above. We predicted that if the swim and scratch networks largely overlap or converge prior to motoneurons, the motoneuron membrane potential would oscillate with one rhythm; if the swim and scratch networks only converge in motoneurons, however, the motoneuron membrane potential would display evidence of two oscillatory inputs. We observed a single, regular oscillation of motoneuron membrane potentials during dual stimulation, with no evidence of a second rhythmic input. These results support the hypothesis that the swim- and scratch-evoking inputs converge and generate a single rhythm prior to motoneurons, in spinal interneurons. To our knowledge, this is the first demonstration of such integration of rhythm-evoking inputs at the interneuronal level for different rhythmic limb movements in adult vertebrates.

## Materials and Methods

### Surgical Procedures

All animal procedures were approved by the Institutional Animal Care and Use Committee of the University of Oklahoma. Adult red-eared turtles *Trachemys scripta elegans*, of both sexes (*n* = 14), weighing 270–570 g, were prepared for recording as described previously (Robertson et al., 1985; Berkowitz, 2001). Briefly, animals were anesthetized by hypothermic analgesia and surgically dissected to (1) transect the spinal cord between the dorsal 2 (D2) and D3 post-cervical segments, (2) expose the spinal cord between the D6 and sacral 2 (S2) segments, and (3) prepare several right hindlimb motor nerves for extracellular recordings and stimulation: the hip flexor (HF), ventral puboischiofemoralis internus, pars anteroventralis; the hip extensor (HE), flexor cruris, pars flexor tibialis internus; and the knee extensors (KEs), triceps femoralis, pars iliotibialis (IT-KE), pars ambiens (AM-KE), and/or pars femorotibialis (FT-KE) (Robertson et al., 1985). After the surgery, turtles were warmed to room temperature for 30 min, then immobilized with gallamine triethiodide (8 mg/kg i.m.; Sigma-Aldrich, St. Louis, MO, United States) and artificially ventilated throughout the experiment. After the experiment, turtles were euthanized by i.p. injection of 1 ml 390 mg/ml pentobarbital (Euthasol; Western Medical Supply, Arcadia, CA, United States).

### Stimulation Procedures

Forward-swimming motor patterns were evoked by electrical stimulation in the D3 contralateral lateral funiculus (0.1-ms, 100–300 μA, bipolar pulses at 10–60 Hz) with a pair of 100-μm silver wires (California Fine Wire, Grover Beach, CA, United States), insulated except at the tips, with one tip contacting the D3 face of the spinal cord and the other in the saline (Lennard and Stein, 1977; Juranek and Currie, 2000; Berkowitz, 2002). The swim stimulation amplitude and frequency were usually adjusted to evoke a swimming motor pattern with a cycle frequency that differed from the scratching cycle frequencies. Rostral, pocket, and caudal scratching motor patterns were evoked by continual gentle rubbing of a single site in the receptive field of each scratch form at ∼3 N, ∼3–4 Hz, using a glass probe with a fire-polished tip (Mortin et al., 1985; Hao et al., 2014). “Swim/scratch dual stimulation” refers to the combination of swim and scratch stimulation delivered at overlapping times.

### Electrophysiology

Dissected nerves were submerged in mineral oil, surrounded by a wax well molded onto the turtle carapace. Recordings from each nerve were obtained extracellularly using a pair of 100-μm silver wires and amplified and filtered (x 1000; band-pass 0.1–1.0 kHz; A-M Systems, Carlsborg, WA, United States); these nerve-recording electrodes were also used to stimulate motoneurons antidromically.

Intracellular recordings (*n* = 21 cells) were obtained from the ipsilateral hindlimb enlargement using sharp electrodes, made by a P-97 puller (Sutter Instrument Company, Novato, CA, United States) and filled with 3 M potassium chloride (Fisher Scientific) or 4 M potassium acetate (Mallinckrodt Baker, Inc., Paris, KY, United States) with resistances of 40–120 MΩ (Robertson and Stein, 1988; Berkowitz, 2005). The meninges were torn at the site of each electrode penetration. Both nerve and single-cell recordings were stored on a digital audio tape recorder (TEAC America, Montebello, CA, United States).

### Antidromic Stimulation

**FIGURE 1:**
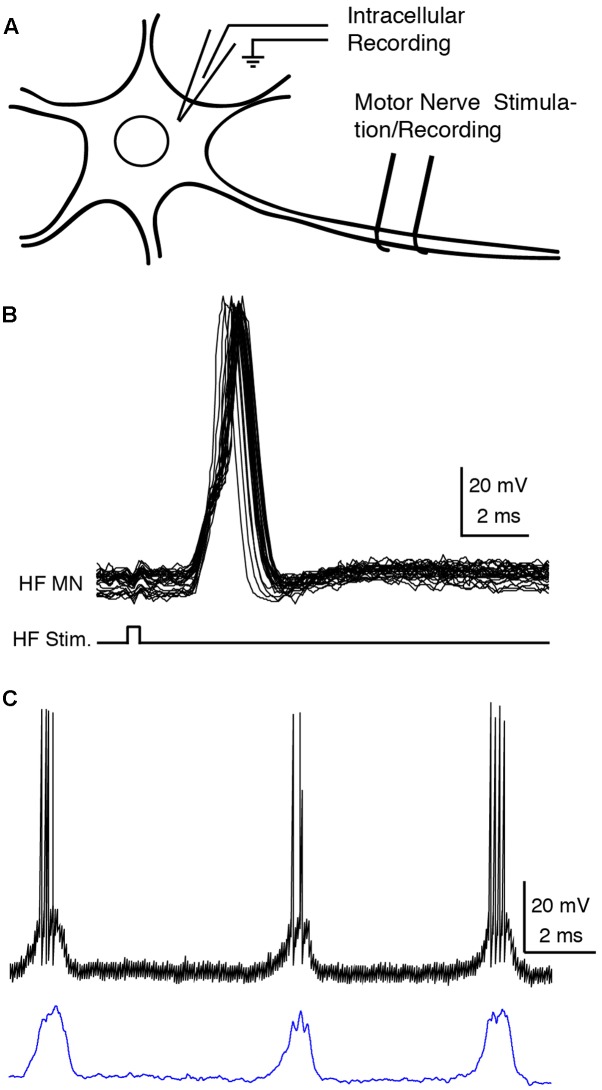
Recording and analysis of motoneurons. **(A)** Experimental design. **(B)** Superimposed traces of the intracellular recording (HF MN, upper traces) during a train of 40-Hz electrical pulses (bottom trace) to the hip flexor (HF) nerve, confirming motoneuron identify by antidromic stimulation. **(C)** Motoneuron recording before (above) and after (below) action potential deletion and smoothing. HF, hip flexor; MN, motoneuron; Stim., stimulation.

One of the dissected nerves was stimulated (0.5–10 V, 0.1-ms, 1–100 Hz or a single pulse) to evoke antidromic action potentials in the intracellularly recorded motoneuron (**Figures 1A,B**). A motoneuron was confirmed when (1) the delay between the stimulation and the onset of the action potential was less than 1 ms with a threshold lower than 2 V (Robertson and Stein, 1988) or (2) the onsets of the action potentials evoked by a train of antidromic stimuli were consistent (Brock et al., 1953).

### Data Analysis

Six cells were not used for quantitative analysis because the recordings were lost before delivery of swim and/or scratch stimulation alone; they behaved qualitatively like the other cells. Recordings of the remaining 15 cells were redigitized and analyzed using Datapac software (Run Technologies, Laguna Hills, CA, United States). Action potential rates were calculated by the action potential count per cycle divided by the cycle duration. Then the action potentials were deleted and the remaining membrane potentials linearly smoothed using a sliding 50-ms window (**Figure 1C**). The summation of swimming and scratching membrane potentials was performed using Matlab (Mathworks, Inc., Natick, MA, United States) by adding the two membrane potentials linearly (Sirois et al., 2013). Six delays (in increments of 0.5 s) were also introduced between the two voltages to be summed (**Figures 3**, **4**, **7**, **10**) in case this significantly affected whether the summed voltage exhibited evidence of two oscillations.

Dual-referent phase-normalized membrane potential averages and their standard deviations (SDs) were calculated by first dividing each cycle into HF-burst (HF-ON) and HF-quiescent (HF-OFF) periods based on the activity of the HF nerve (Berkowitz and Stein, 1994). The membrane potential of each motoneuron was then separately phase-normalized for the HF-ON period and the HF-OFF period (Berkowitz, 2005), because HF duty cycle varies substantially during episodes of swimming and scratching. Dual-referent phase analysis is preferable to single-referent phase analysis whenever duty cycle varies substantially, because single-referent phase analyses can be misleading in such cases (see Figure 2 in Berkowitz and Stein, 1994). Only cycles completely within the period of stimulation were analyzed. HE-phase deletion cycles, defined by the absence of the quiescence between successive HF bursts (Stein and Grossman, 1980; Stein, 2008), were not included in the quantitative analysis. All other cycles were included in the analysis for each neuron.

### Statistics

Statistical comparisons (Instat 3 and Prism 7, GraphPad Software, San Diego, CA, United States) of SDs were made within each form of motor pattern across animals using the repeated-measure non-parametric ANOVA test (Friedman’s test) to test for any statistical significance across the group of SDs as a whole, followed by selected pair comparisons (Dunn’s test), to determine whether these two SDs differed significantly from each other. The multiplicity adjusted *p*-value is reported for statistically significant comparisons using Dunn’s test.

## Results

Electrical stimulation of the contralateral D3 lateral funiculus evoked pure-form forward swimming motor patterns (henceforth, just “swimming”). Mechanical stimulation of the ipsilateral body surface evoked pure-form rostral, pocket, or caudal scratching motor patterns (henceforth, just “scratching”), depending on the stimulation location. Swim and each form of scratch stimulation delivered at an overlapping time period (swim/scratch dual stimulation) could increase the rhythm frequency and/or alter the motor pattern itself (Hao et al., 2011). Here, we intracellularly recorded from 15 hip flexor (HF) motoneurons, 2 hip extensor (HE) motoneurons, and 4 knee extensor (KE) motoneurons (2 AM-KE and 2 FT-KE; see Materials and Methods), which were confirmed by antidromic stimulation of the respective motor nerves (**Figure 1**; see Materials and Methods), and analyzed their responses to the swim/scratch dual stimulation.

### Motoneuron Membrane Potentials during Swim/Rostral Scratch Dual Stimulation

**FIGURE 2:**
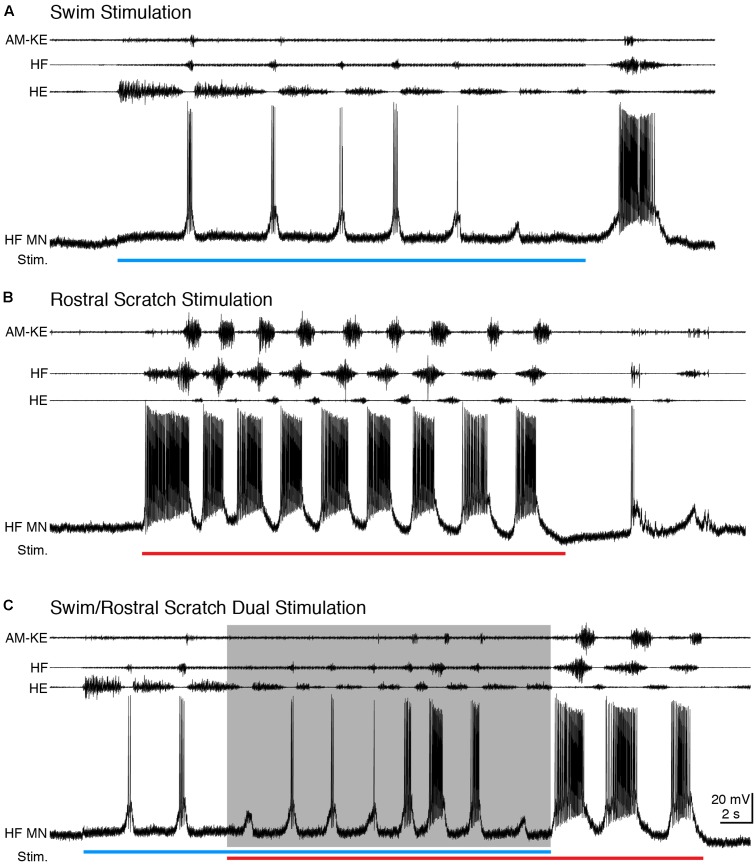
During dual stimulation, this HF motoneuron’s membrane potential still oscillated regularly. **(A–C)** Extracellular recordings from limb nerves (top three traces; AM-KE, ambiens knee extensor; HF, hip flexor; HE, hip extensor) and the intracellular recording of the motoneuron shown in **Figure 1** (HF MN, fourth trace). **(A)** Recordings during swim stimulation [current pulses at 25 Hz; blue bar indicates the stimulation period (Stim.)]. **(B)** Recordings during rostral scratch stimulation (red bar). **(C)** When swim/rostral scratch dual stimulation (blue and red bars) evoked a motor pattern with a higher cycle frequency (shaded area), the membrane potential of the HF motoneuron oscillated regularly at the same rate as the motor pattern.

During swimming, HF and HE nerves were activated rhythmically and alternately, with HF bursts briefer and weaker than HE bursts and AM-KE knee extensor bursts slightly delayed relative to HF bursts (Lennard and Stein, 1977; Juranek and Currie, 2000) (**Figure 2A**). Rostral scratching also featured rhythmic alternation between HF and HE bursts, while the HF bursts were longer and stronger than HE bursts and AM-KE was active approximately in the second half of each HF burst (Robertson et al., 1985) (**Figure 2B**). For all motoneurons studied (*n* = 15), we were able to adjust the swim stimulation parameters to evoke swimming at a substantially different frequency than rostral scratching. During swim stimulation, HF motoneurons displayed brief depolarizations in phase with HF nerve bursts and fired only a few action potentials (mean ± SD: 0.50 ± 0.52 spikes/s, *n* = 13 cycles for example in **Figure 2A**). During rostral scratch stimulation, each HF motoneuron’s membrane potential displayed large and regular oscillations that were in phase with the HF nerve bursts and the motoneuron fired action potentials at a higher rate (37.4 ± 23.6 spikes/s, *n* = 14 cycles for **Figure 2B**) than during swimming (see also Robertson and Stein, 1988). When rostral scratch (cycle frequency: 0.38 ± 0.15 Hz, *n* = 14 cycles) stimulation was added to an ongoing swim (cycle frequency: 0.26 ± 0.13 Hz, *n* = 12) stimulation, the motor pattern cycle frequency typically increased (cycle frequency: 0.46 ± 0.22 Hz, *n* = 12) (**Figure 2C**). The HF motoneuron membrane potential oscillated regularly and in phase with the HF nerve bursts, with an intermediate spike rate (4.97 ± 4.20 spikes/s). After the end of the swim stimulation, the cycle frequency slowed down and the motor pattern became rostral scratch-like. In this example, the motor pattern during the dual stimulation switched from swim-like (HF < HE) toward rostral scratch-like (HF > HE), back to swim-like. Matching the motor pattern, the motoneuron membrane potential gradually switched from swim-like (brief depolarization with few action potentials) toward rostral scratch-like (strong depolarization with many action potentials) and then switched back to swim-like on the last cycle.

In many other cases, the motor pattern could be swim-like during the entire swim/rostral scratch dual stimulation and the motoneuron membrane potential then oscillated regularly and matched the motor patterns (data not shown). Regardless of the motor pattern, the membrane potential oscillated regularly and in phase with the nerve. The motoneuron firing rates during swim, rostral scratch, and dual stimulation were 7.3 ± 4.0, 8.8 ± 3.6, and 8.6 ± 3.7 spikes/s (mean ± SEM) across all neurons with sufficient data (*n* = 15, 10, and 11 cells, respectively).

We reasoned that if the swim and rostral scratch networks are separate and their signals only converge onto motoneurons, the membrane potential oscillations of the motoneurons would show signs of both the swim rhythm and the rostral scratch rhythm. Alternatively, if the swim and scratch signals are integrated prior to motoneurons, we would only observe one rhythmic input to the motoneurons.

**FIGURE 3:**
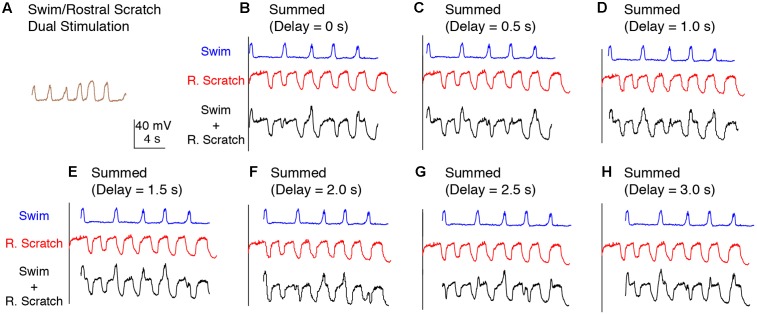
Calculated linear summations of the membrane potential oscillations during swim stimulation alone and rostral scratch stimulation alone did not produce regular oscillations. **(A)** The membrane potential oscillations during swim/rostral scratch dual stimulation shown in **Figure 2C**. **(B–H)** Calculated summations (Swim + R. Scratch, black traces) of the membrane potential oscillations during swim stimulation alone (Swim, blue traces) and rostral scratch stimulation alone (R. Scratch, red traces) at various delays.

To test between these two hypotheses more rigorously, we deleted the action potentials from each intracellular recording and smoothed the remaining membrane potential to better visualize the waveform oscillations (**Figure 1C**; see Materials and Methods). The membrane potential oscillated regularly during swim/rostral scratch dual stimulation (**Figure 3A**). The cycle frequency was higher during dual stimulation than during swim stimulation alone (**Figures 3B–H**, blue traces) and rostral scratch stimulation alone (**Figures 3B–H**, red traces). Then, we linearly summed the membrane potential during swim stimulation alone and scratch stimulation alone with various delays between them (0–3 s, in 0.5-s increments), in case this significantly affected the evidence for two oscillations. Although one would not expect motoneurons to add their inputs linearly, one would expect to see signs of both rhythmic inputs in the motoneuron membrane potential if two different rhythmic inputs occurred simultaneously. The arithmetic addition yielded waveforms that were irregular with additional peaks, troughs, or prolonged cycle durations (**Figures 3B–H**, black traces). In contrast, such irregularities in the membrane potential were never observed during swim/rostral scratch dual stimulation. Also, we never observed an arithmetic summation that generated a consistently increased cycle frequency. Thus, dual stimulation evoked a membrane potential oscillation that was qualitatively different from the summation of two different rhythmic oscillations.

**FIGURE 4:**
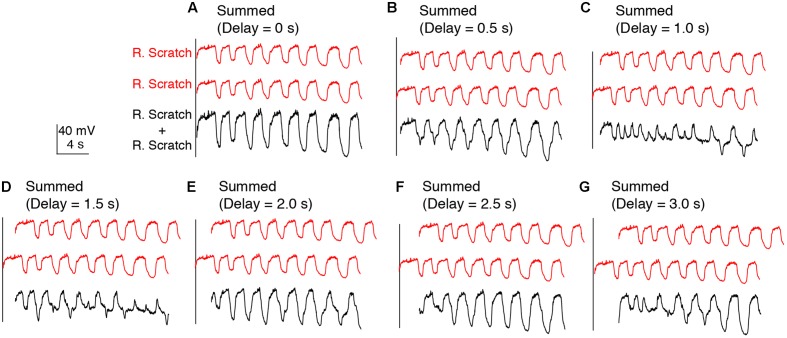
Calculated linear summations of two identical sets of membrane potential oscillations during rostral scratch stimulation alone did not produce regular oscillations, except when the two sets were in phase. **(A–G)** Calculated summations (R. Scratch + R. Scratch, black traces) of two sets of membrane potential oscillations during rostral scratch stimulation alone (R. Scratch, red traces) at various delays.

In addition, we examined the effects of linearly summing two sets of identical oscillations recorded during either rostral scratch stimulation alone (**Figure 4**) or swim stimulation alone (data not shown) with various delays between them. In both cases, this addition generated irregular oscillations with additional peaks and troughs (**Figures 4B–G**), except when the two sets of identical oscillations were precisely in phase (i.e., 0-s delay; **Figure 4A**). Thus, if motoneurons received two sets of rhythmic inputs at overlapping times, irregularities would likely be seen in the motoneuron membrane potential, even if the two sets of oscillations were the same.

**FIGURE 5:**
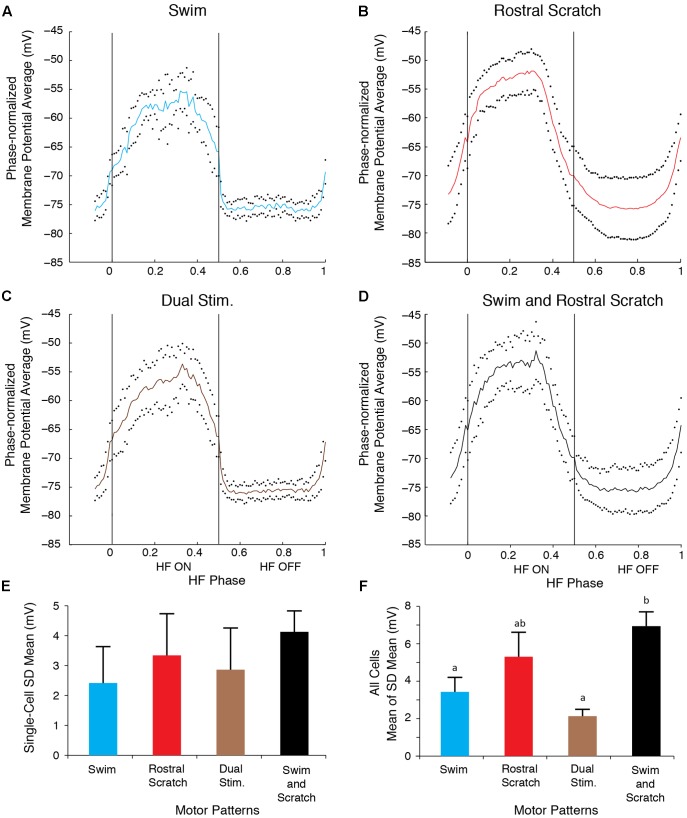
The motoneuron membrane potential oscillations during swim/rostral scratch dual stimulation were as regular as those during swim stimulation alone or rostral scratch stimulation alone. **(A–C)** Dual-referent phase-normalized membrane potential average and the SD (black dots) across all cycles for the cell in **Figure 2** with the same stimulation parameters during swim stimulation (**A**, blue curve), rostral scratch stimulation (**B**, red curve), and swim/rostral scratch dual stimulation (**C**, brown curve). **(D)** Dual-referent phase-normalized membrane potential average of the pooled set of all swim stimulation-alone cycles (from **A**) and all rostral scratch-stimulation alone cycles (from **B**) combined for this cell. **(E)** The mean of the SD within each stimulation paradigm for this cell. Error bars, SD. **(F)** The mean of the mean SD in **(E)** across all 10 cells with the same stimulation paradigm. Error bars, SE. a, b, indicate significant differences (identical letters indicate no statistically significant difference; different letters indicate a significant difference) by Friedman’s test followed by Dunn’s test for *post hoc* pairwise comparisons (Friedman’s test: *p* = 0.003; Dunn’s test: swim stimulation vs. pooled, *p* = 0.047; dual stimulation vs. pooled, *p* = 0.012; other pairs were not significantly different).

To analyze quantitatively the consistency of the membrane potential oscillations during swim/rostral scratch dual stimulation, we also calculated dual-referent phase-normalized membrane potential averages (solid curves) and SDs (dots) with respect to the HF-on and HF-off phases of each cycle (see Materials and Methods). That is, we calculated the SD of the motoneuron membrane potential at each phase of the cycle, averaged across several cycles, as a measure of the consistency of the voltage change across the cycle. In **Figures 5A–E**, we illustrate results of this analysis for one neuron (the same neuron as shown in **Figure 3**). In the cell illustrated, the SD during the swim/rostral scratch dual stimulation (**Figures 5C,E**; *n* = 14 cycles) was greater than during swim stimulation alone (**Figures 5A,E**; *n* = 13 cycles), but was smaller than during rostral scratch stimulation alone (**Figures 5B,E**; *n* = 17 cycles). The SD during dual stimulation (**Figures 5C,E**) was also smaller than the SD obtained by pooling together all cycles of swim stimulation alone (from **Figure 5A**) and scratch stimulation alone (from **Figure 5D**) and calculating the phase-normalized membrane potential average of all these cycles together (**Figures 5D,E**). This shows that the voltage trajectory during dual stimulation is more consistent than for a group of cycles in which some cycles are swim-like and other cycles are rostral scratch-like.

These findings were consistent across the 10 motoneurons for which we collected enough data during swim, rostral scratch, and swim/rostral scratch dual stimulation (**Figure 5F**). The SDs during swim/rostral scratch dual stimulation were not significantly different from SDs during either swim stimulation alone or rostral scratch stimulation alone, but were significantly smaller than the SD of the pooled swim stimulation-alone and scratch stimulation-alone cycles (**Figure 5F**; Friedman’s test, *p* = 0.003, followed by Dunn’s test: swim stimulation vs. pooled, *p* = 0.047; dual stimulation vs. pooled, *p* = 0.012; other groups were not significantly different). The relatively small SDs during swim/rostral scratch dual stimulation suggest that each motoneuron received a single, regular rhythmic input, rather than two different rhythmic inputs. An additional reason for the larger SD in both the rostral scratch stimulation-alone (**Figure 5B**) and the pooled-cycles (**Figure 5D**) analyses may be that the motoneuron voltage varied more during the HE phase across an episode of rostral scratching (e.g., **Figure 2B**) than across an episode of swimming (e.g., **Figure 2A**). The relatively low membrane potential SD during dual stimulation (similar to the SD during swim stimulation alone) is consistent with the motor pattern during dual stimulation being swim-like.

### Motoneuron Membrane Potentials during Swim/Pocket Scratch Dual Stimulation

**FIGURE 6:**
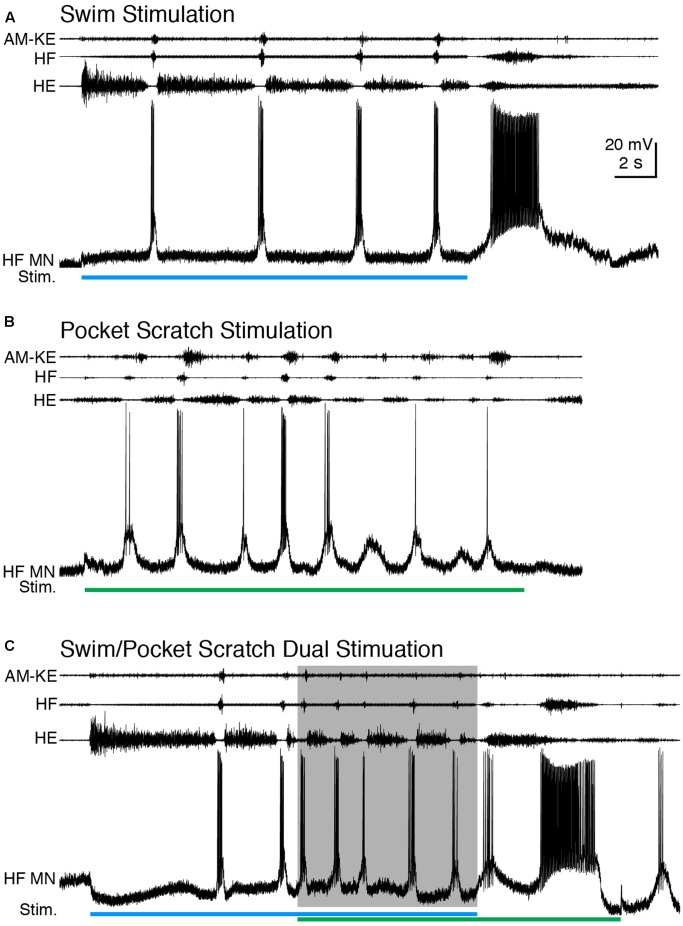
When the cycle frequency was increased by swim/pocket scratch dual stimulation, this HF motoneuron’s membrane potential oscillated regularly. **(A–C)** Extracellular recordings from limb nerves and the intracellular recording of the same HF motoneuron shown in **Figures 1**, **2**. **(A)** Recordings during swim stimulation alone (current pulses at 40 Hz; blue bar). **(B)** Recordings during pocket scratch stimulation alone (green bar). **(C)** When swim/pocket scratch dual stimulation (blue and green bars) evoked a motor pattern with a higher cycle frequency (shaded area), the membrane potential of the HF motoneuron oscillated regularly at the same rate as the motor pattern.

**FIGURE 7:**
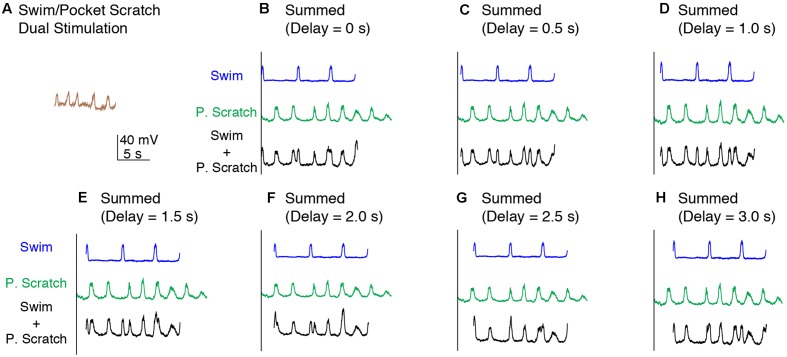
Calculated linear summations of the membrane potential oscillations during swim stimulation alone and pocket scratch stimulation alone did not produce regular oscillations. **(A)** The membrane potential oscillations during swim/pocket scratch dual stimulation shown in **Figure 6C**. **(B–H)** Calculated summations (Swim + P. Scratch, black traces) of the membrane potential oscillations during swim stimulation alone (Swim, blue traces) and pocket scratch stimulation alone (P. Scratch, green trace) at various delays.

We also applied dual swim/scratch stimulation using pocket scratch and caudal scratch stimuli. For 12 of the 15 motoneurons we studied while evoking pocket scratching, we were able to adjust the swim stimulation parameters to evoke swimming at a substantially different frequency than pocket scratching. Pocket scratching featured rhythmic alternation between HF and HE nerve bursts with the AM-KE and FT-KE nerve bursts continuing during part of the HE nerve bursts (Robertson et al., 1985) (**Figure 6B**). During swim/pocket scratch dual stimulation, the motor pattern was swim-like, with an AM-KE nerve burst at the end of each HF nerve burst. The depolarization phase of the motoneuron membrane potential was swim-like while the hyperpolarization phase was less consistent, like that during pocket scratch stimulation (**Figure 6C**). The membrane potential oscillation during the swim/pocket scratch dual stimulation was regular (**Figure 7A**) whereas the calculated addition of the membrane oscillation during swim stimulation alone (**Figures 7B–H**, blue traces) and pocket scratch stimulation alone (**Figures 7B–H**, green traces) at various delays created double bursts or prolonged excitations (**Figures 7B–H**, black traces).

The cycle frequencies during swimming, pocket scratching, and dual stimulation were 0.18 ± 0.06 Hz (*n* = 4 cycles), 0.40 ± 0.17 Hz (*n* = 10), and 0.53 ± 0.12 Hz (*n* = 4), respectively. Thus, cycle frequency increased during dual stimulation. The motoneuron firing rates during swim, pocket scratch, and dual stimulation were 2.5 ± 1.9, 0.9 ± 1.2, and 3.5 ± 1.7, respectively, for the neuron shown in **Figure 6** and 7.4 ± 3.9, 14.9 ± 5.0, and 20.0 ± 14.6 spikes/s, respectively, for all neurons with sufficient data (*n* = 15, 14, and 14, respectively).

**FIGURE 8:**
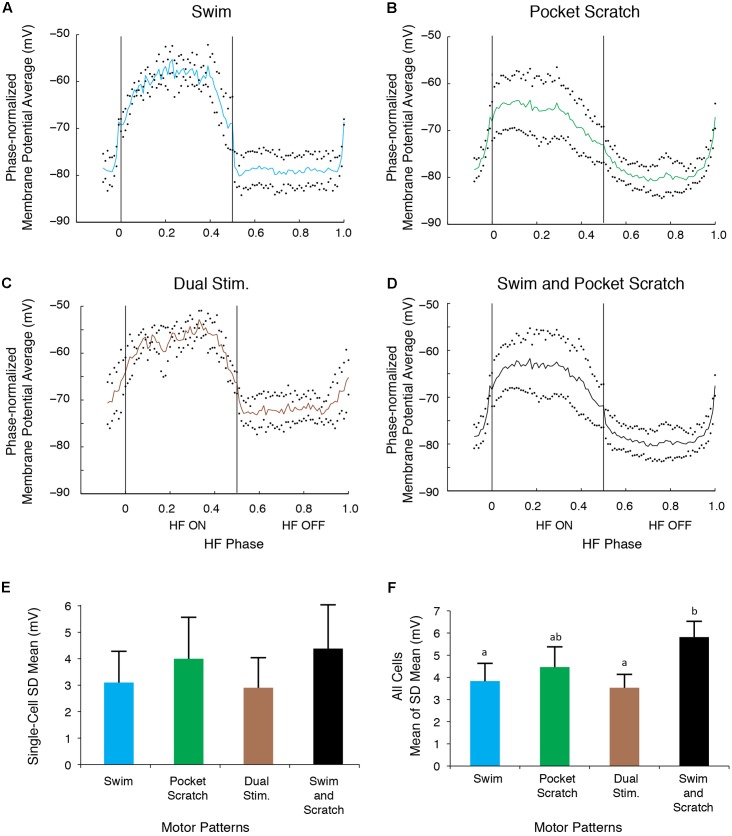
The motoneuron membrane potential oscillations during swim/pocket scratch dual stimulation were as regular as those during swim stimulation alone or pocket scratch stimulation alone. **(A–C)** Dual-referent phase-normalized membrane potential average and the SD (black dots) across all cycles of the cell in **Figure 6** with the same stimulation parameters during swim stimulation (**A**, blue curve), pocket scratch stimulation (**B**, green curve), and swim/pocket scratch dual stimulation (**C**, brown curve). **(D)** Dual-referent phase-normalized membrane potential average of the pooled set of all swim stimulation-alone cycles (from **A**) and all pocket scratch stimulation-alone cycles (from **B**) for this cell. **(E)** The mean of the SD within each stimulation paradigm. Error bars, SD. **(F)** The mean of the mean SD in **(E)** across all 13 cells with the same stimulation paradigm. Error bars, SE. a, b, indicate significant differences by Friedman’s test followed by Dunn’s test (Friedman’s test: *p* = 0.017; Dunn’s test: swim stimulation vs. pooled, *p* = 0.031; dual stimulation vs. pooled, *p* = 0.031; other pairs were not significantly different).

The SD during the swim/pocket scratch dual stimulation for the cell shown in **Figures 6**, **7** (**Figures 8C,E**, brown bar; *n* = 4 cycles) was smaller than or equal to those during swim stimulation alone (**Figures 8A,E**; *n* = 3 cycles), pocket scratch stimulation alone (**Figures 8B,E**; *n* = 7 cycles), and all cycles pooled from both swim stimulation alone and pocket scratch stimulation alone (**Figures 8D,E**). Collectively, for the 13 cells we were able to test, the SDs during swim/pocket scratch dual stimulation were not significantly different from SDs during either swim stimulation alone or pocket scratch stimulation alone, but were significantly smaller than the SD of the pooled swim stimulation-alone and scratch stimulation-alone cycles (**Figure 8F**; Friedman’s test, *p* = 0.017, followed by Dunn’s test: swim stimulation vs. pooled, *p* = 0.031; dual stimulation vs. pooled, *p* = 0.031; other groups were not significantly different). This suggests that swim/pocket scratch dual stimulation also evokes a single rhythmic input to motoneurons.

### Increased Cycle Frequency during Swim/Caudal Scratch Stimulation

**FIGURE 9:**
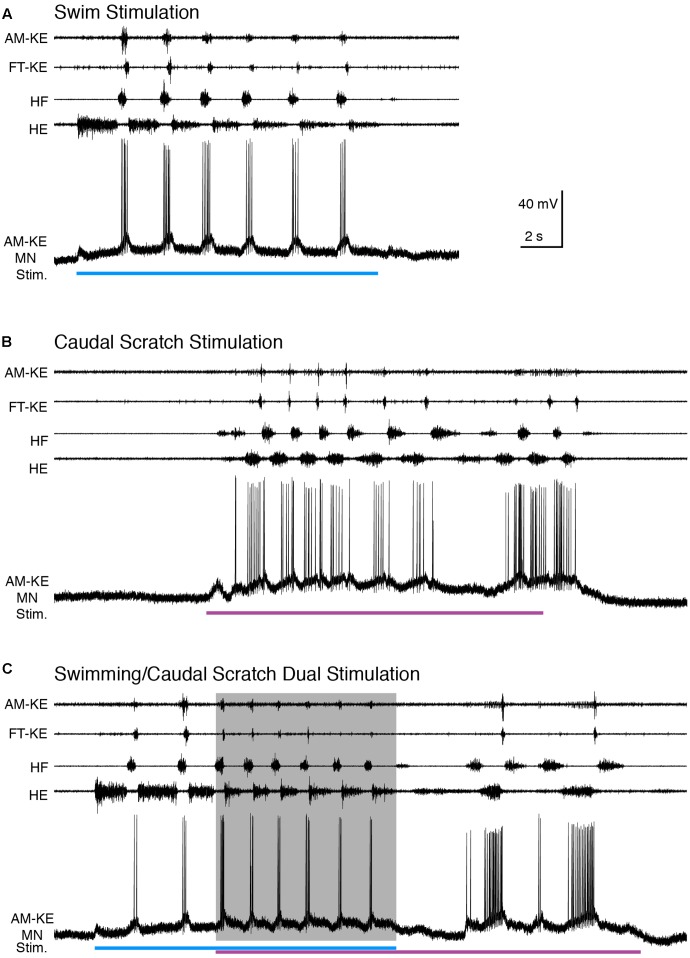
When the cycle frequency was increased by swim/caudal scratch dual stimulation, this AM-KE motoneuron’s membrane potential oscillated regularly. **(A–C)** Extracellular recordings from limb nerves (top four traces; AM-KE, ambiens knee extensor; FT-KE, femorotibialis knee extensor; HF, hip flexor; HE, hip extensor) and the intracellular recording of an AM-KE motoneuron. **(A)** Recordings during swim stimulation alone (current pulses at 40 Hz, blue bar). **(B)** Recordings during caudal scratch stimulation alone (purple bar). **(C)** When swim/caudal scratch dual stimulation (blue and purple bars) evoked a motor pattern with a higher cycle frequency (shaded area), the membrane potential of the AM-KE motoneuron oscillated regularly at the same rate as the motor pattern.

Caudal scratching featured rhythmic alternation between HF and HE bursts with the HF bursts weaker and briefer than the HE bursts (Robertson et al., 1985) (**Figure 9A**). FT-KE bursts occurred at approximately opposite phases for swimming (end of each HF burst; **Figure 9A**) and scratching (start of each HF burst; **Figure 9B**). For all motoneurons studied (*n* = 15), we were able to adjust the swim stimulation parameters to evoke swimming at a substantially different frequency than caudal scratching. **Figure 9** shows an example of an AM-KE motoneuron recording.

Compared to the brief and regular depolarization of this motoneuron during swim stimulation, the depolarization of this AM-KE motoneuron during caudal scratch stimulation was prolonged. During swim/caudal scratch dual stimulation, the motor pattern was swim-like with the AM-KE nerve burst at the end of each HF burst. The depolarizing phases of the membrane potential were sharp and swim-like, but the membrane potential declined only gradually from its peak depolarization and in this respect was caudal scratch-like (**Figure 9C**).

The cycle frequencies during swim, caudal scratch, and dual stimulation were 0.42 ± 0.06 Hz (*n* = 7 cycles), 0.54 ± 0.14 Hz (*n* = 18), and 0.59 ± 0.09 Hz (*n* = 21), respectively. Thus, cycle frequency increased during dual stimulation. The motoneuron firing rates during swim, caudal scratch, and dual stimulation were 4.1 ± 2.5, 1.2 ± 1.0, and 1.8 ± 0.6 spikes/s, respectively, for the neuron shown in **Figure 9** and 7.3 ± 4.0, 9.9 ± 4.2, and 6.1 ± 1.7, respectively, for all neurons with sufficient data (*n* = 15, 9, and 9, respectively).

**FIGURE 10:**
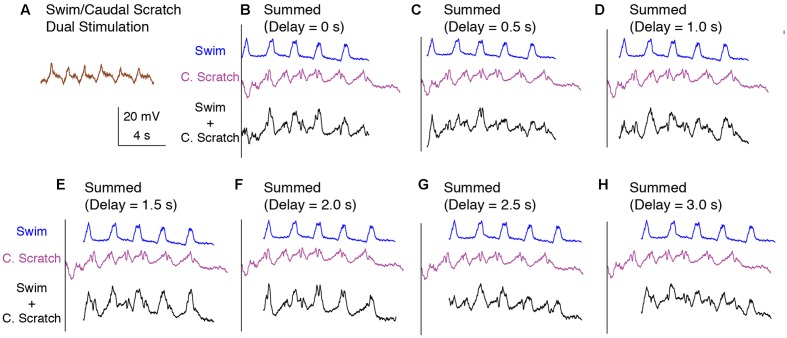
Calculated linear summations of the membrane potential oscillations during swim stimulation alone and caudal scratch stimulation alone did not produce regular oscillations. **(A)** The membrane potential oscillations during swim/caudal scratch dual stimulation shown in **Figure 9C**. **(B–H)** Calculated summations (Swim + C. Scratch, black traces) of the membrane potential oscillations during swim (Swim, blue traces) and caudal scratch (C. Scratch, purple traces) stimulation alone at various delays.

The membrane potential oscillation during swim/caudal scratch dual stimulation was regular (**Figure 10A**) whereas the calculated addition of the membrane oscillations during swim stimulation alone (**Figures 10B–H**, blue traces) and caudal scratch stimulation alone (**Figures 10B–H**, purple traces) at various delays created irregular oscillations with an oscillation amplitude that varied from cycle to cycle.

**FIGURE 11:**
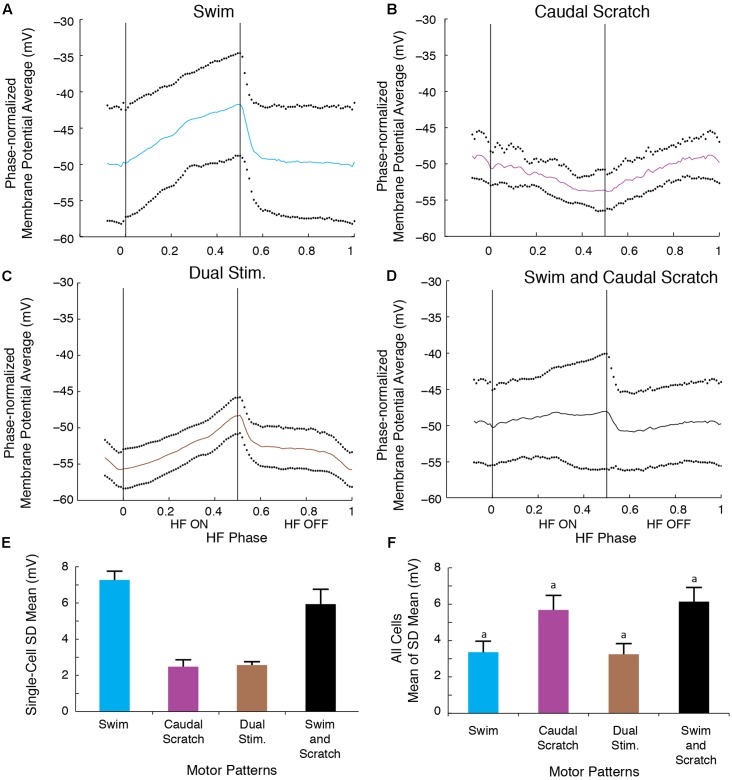
The motoneuron membrane potential oscillations during swim/caudal scratch dual stimulation were as regular as those during swim or caudal scratch stimulation alone. **(A–C)** Dual-referent phase-normalized membrane potential average and the SD (black dots) across all cycles of the cell in **Figure 9** with the same stimulation parameters during swim stimulation (**A**, blue curve), caudal scratch stimulation (**B**, purple curve), and swim/caudal scratch dual stimulation (**C**, brown curve). **(D)** Dual-referent phase-normalized membrane potential average of the pooled set of all swim stimulation-alone cycles (from **A**) and all caudal scratch stimulation-alone cycles (from **B**) for this cell. **(E)** The mean of the SD within each stimulation paradigm for this cell. Error bars, SD. **(F)** The mean of the mean SD in **(E)** across all eight cells with same stimulation paradigm. Error bars, SE. ^a^Although the distribution of data as a whole was significantly different from random (Friedman’s test, *p* = 0.013), *post hoc* pairwise comparisons (Dunn’s test) showed no significant differences (*p* > 0.05) among the tested pairs.

The SD of the phase-averaged membrane potential during the swim/caudal scratch dual stimulation for this cell (**Figures 11C,E**; *n* = 11 cycles) was similar to the SD during caudal scratch stimulation alone (**Figure 11B,E**; *n* = 11 cycles), but less than the SD during swim stimulation alone (**Figures 11A,E**; *n* = 13 cycles) and all cycles pooled from both swim and caudal scratch stimulation alone (**Figures 11D,E**). Collectively, for the eight cells for which we had sufficient data, the SDs during swim/caudal scratch dual stimulation were not significantly different from the SDs in each of the three other categories, although the distribution of data as a whole was significantly different from random (**Figure 11F**; Friedman’s test, *p* = 0.013 followed by Dunn’s test, *p* > 0.05 for each single comparison).

### Altered Motor Patterns

**FIGURE 12:**
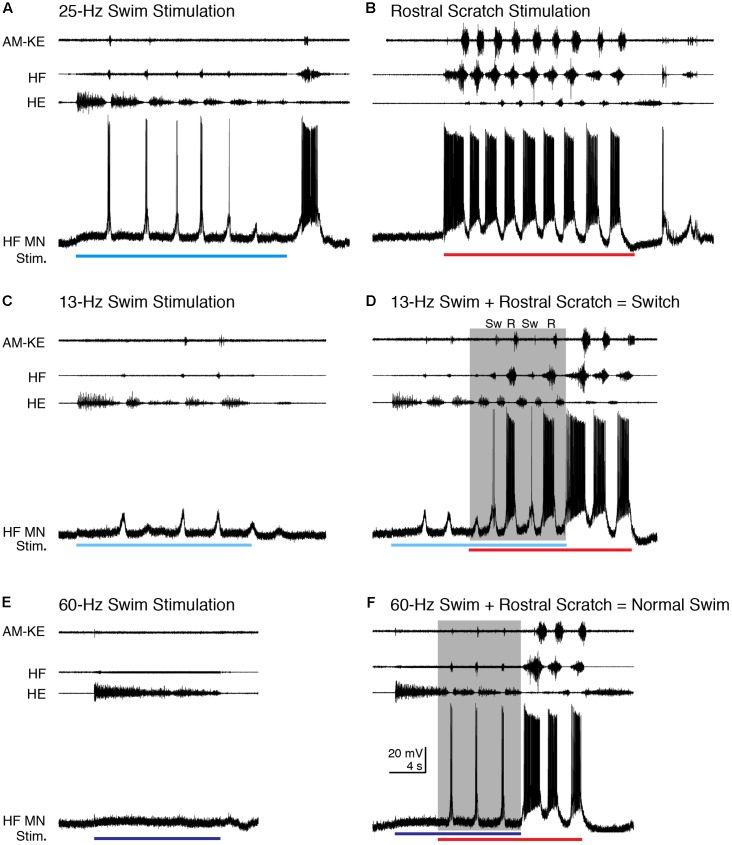
The motoneuron membrane potential matched nerve outputs when scratch inputs altered the motor pattern evoked using different swim stimulation frequencies. **(A)** Typical swimming evoked by 25-Hz swim stimulation (blue bar). **(B)** Typical rostral scratching evoked by rostral scratch stimulation (red bar). **(C)** Weaker swimming evoked by 13-Hz swim stimulation (light blue bar). Notice the motor pattern was still swim-like although this motor neuron remained subthreshold for action potentials. **(D)** When rostral scratch stimulation was added to the 13-Hz swim stimulation, the motor pattern switched between swim-like (Sw) and rostral scratch-like (R). After the end of the swim stimulation, the rostral scratch stimulation alone evoked a typical rostral scratch motor pattern and motoneuron membrane potential oscillation. **(E)** An overly high-frequency swim stimulation (60 Hz, dark blue bar) failed to evoke any swim rhythm. Note that there were no membrane potential oscillations in the HF motoneuron either. **(F)** Adding rostral scratch stimulation to the overly high-frequency swim stimulation (shaded area) evoked a swim-like motor pattern similar to typical swimming shown in **(A)**. The membrane potential was also swim-like during the dual stimulation. The motor pattern and membrane potential changed to rostral scratch-like when the swim stimulation ended and the rostral scratch stimulation continued.

In addition to increasing the cycle frequency, swim/scratch dual stimulation can alter the motor pattern, depending on the stimulation parameters (Hao et al., 2011). One of the effects is that the motor pattern can switch between swim-like and scratch-like within one episode. For two HF motoneurons that we were able to record from for an especially long time, we were able to vary swim stimulation parameters to elicit switches between swimming and scratching. **Figure 12** shows an example for the cell shown in **Figure 2**. During typical swimming evoked by 25-Hz electrical pulses (**Figure 12A**), AM-KE bursts, when they occurred, were very weak, the HF nerve bursts were much weaker and briefer than the HE bursts, and the depolarizations of the HF motoneuron were also weak and triggered only a few action potentials (0.50 ± 0.52 spikes/s, *n* = 13 cycles). During rostral scratch stimulation alone, the AM-KE nerve bursts were strong, the HF nerve bursts were much stronger and longer than the HE bursts (**Figure 12B**), and the depolarizations of the HF motoneuron were also stronger and triggered more action potentials (37.4 ± 23.6 spikes/s). A 13-Hz swim stimulation evoked weaker swimming than 25-Hz stimulation did and the depolarization of the recorded HF motoneuron was subthreshold (**Figure 12C**). When rostral scratch stimulation was added to this weak swim stimulation, the motor pattern switched between swim-like and scratch-like (**Figure 12D**). During this dual stimulation, the spike rate of the intracellularly recorded motoneuron was 0, 1.1, and 0.6 spikes/s, respectively, for the three cycles identified as swim-like (Sw) and 33.7 and 39.1 spikes/s, respectively, for the two cycles identified as rostral scratch-like (R). When the swim stimulation ceased, the motor pattern stayed rostral scratch-like until the end of the rostral scratch stimulation and the intracellularly recorded motoneuron had a spike rate of 68.0 and 46.0 spikes/s, respectively, for these rostral scratch-like cycles. During all of the switching, during and after stimulation, the membrane potential of the HF motoneuron matched the motor output and showed no sign of summing two distinct inputs.

In other cases (*n* = 2 cells), an overly high-frequency swim stimulation could evoke tonic HE activity with no HF bursts (**Figure 12E**; see also Hao et al., 2011). During this stimulation, the membrane potential of the HF motoneuron depolarized slightly without any noticeable oscillation, showing that the motoneuron was not receiving rhythmic inputs (**Figure 12E**). Adding the rostral scratch stimulation to this same swim stimulation evoked the typical swim-like motor pattern with HE-biased HF-HE alternation (**Figure 12F**). During this swim-like motor pattern, the HF motoneuron depolarized regularly and briefly as during normal swimming (**Figure 12F**), with a spike rate of 2.0 ± 0.6 spikes/s. When the overly strong swim stimulation stopped and rostral scratch stimulation continued, both the motor pattern and the HF motoneuron membrane potential oscillations immediately changed to rostral scratch-like for two cycles, with a spike rate of 39.5 and 15.7 spikes/s, in the intracellularly recorded motoneuron. Thus, in this situation also, the rhythmic inputs (or lack of them) to motoneurons matched the nerve outputs. Similar results were seen during swim/pocket scratch stimulation in the same cell (data not shown).

## Discussion

In a previous study, we showed that swim/scratch dual stimulation can evoke motor patterns that differ from those evoked by swim stimulation alone and scratch stimulation alone in both rhythm frequency and motor pattern (Hao et al., 2011). These results demonstrated sharing and/or strong interactions between swimming and scratching networks in both rhythm and pattern generation. However, this previous study only recorded from motor nerves (extracellularly) and thus could not assess whether motoneurons themselves contribute to integrating two inputs to generate a different motor rhythm/pattern or if all interactions that alter the rhythm and/or pattern occur among interneurons instead. Motoneurons might integrate separate swimming and scratching oscillatory inputs, resulting in irregular motoneuron membrane potential oscillations, yet generate action potentials rhythmically because only the peaks of the oscillations (which occur regularly) are suprathreshold. This would be consistent with spinal motoneurons contributing to shaping rhythmic output via active membrane properties (Hounsgaard and Kiehn, 1989; Kiehn et al., 2000; Heckman et al., 2008; Hultborn et al., 2013) and via gap junctions with premotor interneurons (Song et al., 2016). In this study, we recorded intracellularly from motoneurons to monitor the inputs they receive during dual stimulation. This allowed us to assess whether separate swimming and scratching rhythms are integrated by motoneurons or, alternatively, if swim- and scratch-evoking signals are integrated to generate one rhythm prior to motoneuron inputs.

### Swimming and Scratching Inputs Could Affect Each Other’s Rhythm Generation Prior to Motoneurons

During swim/scratch dual stimulation, we often observed a motor pattern that was faster than either swimming or scratching alone (Hao et al., 2011) (**Figures 2**, **6**, **9**). This demonstrates that swimming and scratching inputs interact when generating a rhythm. Here, we recorded intracellularly from motoneurons during dual stimulation and observed regular motoneuron oscillations with a standard deviation similar to pure-form swimming (**Figures 5**, **8**, **11**). The regularity of the membrane potential oscillations during swim/scratch dual stimulation is consistent with the hypothesis that the interaction between swimming and scratching rhythm generation occurs prior to motoneurons.

### The Regularity of Motoneuron Membrane Potential Oscillations Suggests that Motoneurons Do Not Integrate Two Different Rhythmic Inputs

The intracellular recordings during dual stimulation displayed regular oscillations (**Figures 2C**, **6C**, **9C**, **12D,F**), indicative of a single, rhythmic input. In contrast, calculated summations of oscillations from swim stimulation alone and scratch stimulation alone were irregular and had additional peaks, troughs, or prolonged excitation (**Figures 3**, **7**, **10**). Potential confounding factors are that the cell’s electrical resistance, driving force for each ion, and contribution of active membrane properties would be expected to change during the membrane potential oscillation, which would be expected to make the summation of two rhythmic inputs non-linear in any case (Kolind et al., 2012; Vestergaard and Berg, 2015; Petersen and Berg, 2016). Nonetheless, the predicted irregularity of the membrane potential oscillation, if there were two overlapping rhythmic inputs, should still be evident, even if the oscillation amplitudes differ from a linear summation. Thus, the linear summation of two inputs should still provide an informative comparison qualitatively.

The irregularities we observed in the calculated summations of two separate oscillations were probably largely due to differences between the swimming and scratching cycle frequencies. The results of our calculated summations appear very similar to the actual voltage trajectories generated by simultaneous activation of two separate networks (Ramirez and Pearson, 1988; Hennig, 1990). In contrast to this, we observed a single, regular oscillation during swim/scratch dual stimulation, which is consistent with the hypothesis that the pathways for swimming and scratching converge prior to motoneurons, resulting in a single rhythmic input to motoneurons during dual stimulation. Alternatively, it is possible that if the swimming and scratching rhythms had the same cycle frequency during these recordings, the combination of two rhythmic inputs to motoneurons could still be regular if these two oscillations happened to be in phase (e.g., **Figure 4A**). However, we do not think this is likely for the vast majority of our experiments because for 12 of the 15 cells analyzed, we were able to adjust the swim stimulation parameters so that the cycle frequency was substantially different during swim stimulation alone and scratch stimulation alone for each form of scratching tested; for the remaining three cells, the swim frequency was similar only to the pocket scratching frequency. In addition, the frequency of the single rhythmic input during dual stimulation was often higher than the frequency during either stimulation alone.

Some cat spinal motoneurons (Wienecke et al., 2015) and neonatal rat spinal motoneurons and interneurons (Le Gal et al., 2016) receive simultaneous respiratory and locomotor oscillatory inputs that can be coupled, but two separate rhythms remain evident in their membrane potentials, in contrast to our results. Thus, spinal interneurons in rhythm-generating networks may generally coordinate multiple rhythmic inputs but only in some cases integrate them into a single rhythm.

### Swimming and Scratching Inputs Could Affect Motor Pattern Selection Prior to Motoneurons

During switches between swimming and scratching, the motoneuron membrane potentials also oscillated regularly and switched quickly between swim-like and scratch-like (**Figure 12D**). In switches, the membrane potential during each cycle was either swim-like or scratch-like, matching the motor output. Similar switches have been reported in other systems with partly shared networks (Mortin et al., 1985; Stein et al., 1986; Jing and Weiss, 2001). Switches between two motor patterns have also been reported for motor patterns generated by separate networks. But these switches tended to have longer delays (Heitler, 1985; Hennig, 1990) or irregular motoneuron membrane potentials (Heitler, 1985).

In other cases, we observed that overly high-frequency swim stimulation, which evoked tonic HE activity by itself, when combined with scratch stimulation could evoke normal swimming (Hao et al., 2011) (**Figures 12E,F**). During overly high-frequency swim stimulation alone, the motoneuron membrane potential remained relatively flat and showed no sign of oscillations. During dual stimulation, the membrane potential was swim-like and matched the motor output. This result argues against the possibility that during the high-frequency swim stimulation, motoneurons received subthreshold swim-like input, and scratch input merely provided tonic excitation of the motoneurons to make the existing swim oscillations suprathreshold. Rather, this result suggests that the scratch input converged with swim input prior to motoneurons and affected swim rhythm generation at the interneuronal level.

Previous work suggested that gain control mechanisms in turtle spinal cord circuitry compensate for inputs that are otherwise too weak or too strong, keeping the circuitry in an intermediate state of activity in which it operates best (Petersen and Berg, 2016). In our experiment, however, swim stimulation at a higher frequency than that which evoked normal swimming evoked no oscillations at all (see also Hao et al., 2011), suggesting that the system was unable to compensate in this situation. Nonetheless, the addition of scratch stimulation to this high-frequency swim stimulation (which should have evoked even higher activity in the circuitry) did evoke regular oscillations, suggesting that the integration of two different kinds of inputs can be more conducive to producing regular oscillations than an overly strong stimulus of just one kind.

### Persistent Inward Currents Are Unlikely to Account for These Findings

Various ion channels may shape membrane potential oscillations and even motor pattern selection (Li, 2015). For example, L-type Ca^2+^ channels may induce bistable plateau phases that outlast the depolarizing current in turtle spinal cord slices (Hounsgaard and Kiehn, 1989; Alaburda et al., 2002). Both swim and scratch stimulation might enhance such plateau phases (Delgado-Lezama et al., 1997; Alaburda and Hounsgaard, 2003), which could play a role in integrating swimming and scratching synaptic inputs during swim/scratch dual stimulation. However, these plateau potentials are overwhelmed by the high synaptic conductance during scratching (Alaburda et al., 2005) and swimming (Guzulaitis et al., 2016) *in vitro*. Similarly, in our experiments, swim/scratch dual stimulation should have increased synaptic conductances dramatically, so it is unlikely that motoneuron plateau properties by themselves smoothed irregular inputs enough to remove all evidence of two rhythmic inputs.

### Partly Shared Networks

Our results from intracellular recording during dual stimulation are consistent with strong interactions and/or shared components at the spinal interneuronal level for generating swimming and scratching. Studies involving single-interneuron recordings during sequentially evoked rhythmic motor patterns are also generally consistent with shared spinal interneurons for generation of swimming and scratching (Berkowitz, 2002, 2005, 2008, 2010), as well as for generation of other distinct rhythmic motor patterns (Soffe, 1993; Li et al., 2007; Liao and Fetcho, 2008; Geertsen et al., 2011; Trejo et al., 2015). Collectively, these studies make it unlikely that the swimming and scratching pathways are completely separate prior to motoneurons. However, earlier studies also suggest that there are some differences between the spinal interneuronal networks for two rhythmic behaviors (Ritter et al., 2001; Berkowitz, 2002, 2008; Li et al., 2007; McLean et al., 2007; Liao and Fetcho, 2008; McLean and Fetcho, 2008; Satou et al., 2009; Frigon and Gossard, 2010; Mui et al., 2012; Hao et al., 2014). Combining these findings with the current findings, one can conclude that to the extent that there are swim and/or scratch-specialized spinal interneurons that contribute to rhythm and/or pattern generation, they appear to have their effects predominately or exclusively on interneurons, not motoneurons.

## Author Contributions

Z-ZH and AB conceived and designed the research. Z-ZH performed the experiments. Z-ZH and AB analyzed the data. Z-ZH and AB interpreted the results of the experiments. Z-ZH drafted the manuscript. Z-ZH and AB edited and revised the manuscript. All authors have approved the final version of the manuscript and agree to be accountable for all aspects of the work. All persons designated as authors qualify for authorship, and all those who qualify for authorship are listed.

## Conflict of Interest Statement

The authors declare that the research was conducted in the absence of any commercial or financial relationships that could be construed as a potential conflict of interest.
